# The Role of Teachers’ Constructivist Beliefs in Classroom Observations: A Social Cognitive Theory Perspective

**DOI:** 10.3389/fpsyg.2022.904181

**Published:** 2022-06-10

**Authors:** Po-Hsi Chen, Jon-Chao Hong, Jian-Hong Ye, Ya-Jiuan Ho

**Affiliations:** ^1^Department of Educational Psychology and Counseling, National Taiwan Normal University, Taipei City, Taiwan; ^2^Institute for Research Excellence in Learning Sciences, National Taiwan Normal University, Taipei City, Taiwan; ^3^Department of Industrial Education, National Taiwan Normal University, Taipei City, Taiwan; ^4^Faculty of Education, Beijing Normal University, Beijing, China; ^5^Taipei City Government, Taipei City, Taiwan

**Keywords:** classroom observation activities, constructive beliefs in teaching, continuous intention, educational policy, psychological stress, value perception

## Abstract

Previous studies have focused on individuals learning from observing a model to be able to produce the modeled behavior. However, there is a lack of studies emphasizing the perspective of being observed to understand the role of perceived value and stress when teachers act as a teaching model. To address this gap, the present study explored the correlates between teachers’ teaching beliefs, perceived value, psychosocial stress, and continuous intention to be the observed teaching model in classroom observations. Data of 349 respondents were usefully collected, and confirmatory factor analysis with structural equation modeling was performed. Results showed that teachers’ constructivist belief in teaching was positively related to perceived value of being observed and getting feedback, but was negatively related to psychosocial stress. Perceived value was positively related to continuous intention to be observed in future classroom observations, but perceived psychosocial stress was not significantly related to continuous intention. The results of this study can be applied to encourage those who are resistant to presenting their teaching experience in classroom observations.

## Introduction

Bandura’s social cognitive theory (SCT) emphasizes the importance of observational learning (Bandura, 2001). Bandura suggested that in order to engage in observational learning, an individual must have a role model; the individual cognitively perceives learning value that triggers their motivation to continue learning. In social cognitive learning, learners can identify the characteristics of the model behavior and compare the characteristics of the model with their own existing abilities; the observer can then perceive that learning occurs (Martinez et al., 2016). In classroom observation activities, the participant observer provides feedback to the observer and the observer accurately grasps the teaching and learning essentials, thus improving the quality of teaching and learning (e.g., Martinez et al., 2016; Bruns et al., 2018), as well as improving students’ ability development and learning (Bacher-Hicks et al., 2019). As a result, observation activities in the classroom are increasingly being evaluated in teaching practice (Bruns et al., 2018). Moreover, classroom observation activities can sometimes be counterproductive when observers identify instructional improvement issues, and can even make teachers resistant to classroom observation activities (Lam, 2001; Bruns et al., 2018). However, few studies have focused on understanding teachers’ own perceptions of being observed during these activities (Bacher-Hicks et al., 2019). Therefore, this study was conducted to understand teachers’ perceptions of classroom observation activities from the perspective of being observed.

In Taiwan, classroom observation is a very important activity for professional growth in K-12 education. For example, the Ministry of Education (2014) stipulates the requirement of classroom observation to continuously improve the quality of teaching and student learning Therefore, teachers in K-12 education are enforced to participate in classroom observation and being observed. However, Saito and Atencio (2013), in found that teachers in Asian countries were so afraid of being observed by their peers that even some teachers were willing to open their classrooms, they did not discuss teaching practices in depth, and resulting themselves lack social cognitive learning. Moreover, when teachers are observed in the classroom, saving social face is embedded in Confucian ethics (Sum and Kwon, 2020). That is, in terms of the third person effect, teachers may first want to be recognized for their performance in a secular way, and may also feel uncomfortable and even psychologically burdened by worrying that others may judge them poorly. Therefore, Confucianism may be a unique context that can be used to examine the effect of classroom observation activities in Taiwan. However, few studies have focused on the Confucian cultural background to explore how it can influence the beliefs and perceptions of teachers in Taiwan when being observed in the classroom. It is therefore interesting to explore how teachers perceived being observed in Taiwan classroom observation under Confucian culture.

Bandura et al. (1977) proposed a social cognitive framework of triadic reciprocal causation of personal beliefs, behavior, and environment. In a reciprocal interaction model, perceived value helps individuals focus and sustain their efforts to complete tasks (Schiavo et al., 2019). When learners observe and evaluate their learning, perceived value motivates them to put more effort into accomplishing their learning goals (Schunk and DiBenedetto, 2020). Additionally, previous research has explored how positive or negative factors can lead to changes in behavior. For example, in the third-party effect, the observer identifies improvements in the observed person and also puts pressure on the observed person (Locke and Latham, 2015; Conson et al., 2017). However, when teachers are exposed to being observed, the social and emotional climate they and their colleagues create in their classrooms affect their performance (Gold and Windscheid, 2020). Therefore, drawing on social cognitive theory, this study aimed to explore the correlates between teachers’ beliefs and their perceived value of being a teaching model in classroom observation (hereafter, perceived value) and the psychological stress of being observed (hereafter, psychosocial stress), which will contribute to the effectiveness of classroom observation activities in a Confucian society.

## Theoretical Background

### Teachers’ Constructivist Beliefs

In most studies of teachers’ beliefs about teaching, two types of beliefs are categorized: (1) subject- or teacher-oriented beliefs; and (2) constructivist-oriented beliefs (Boulton-Lewis et al., 2001; Meirink et al., 2009; Valckx et al., 2021). The former emphasizes the relevance of content and pedagogy with a more didactic approach, while the latter emphasizes inquiry-oriented learning with more constructivist teaching (Hong et al., 2019a). The constructivist-oriented belief posits student-centered teaching that involves teaching students how to learn, and focuses on the construction of knowledge by students themselves (e.g., Waeytens et al., 2002; Bolhuis and Voeten, 2004). Teachers’ beliefs influence their teaching attitudes and behaviors (Roose et al., 2019), for example, their classroom performance (Hong et al., 2019a). In line with this, the present study considered constructivist teaching beliefs as an essential factor that may affect teachers’ performance as a teaching model in classroom observations, and to investigate whether this teaching belief is an antecedent to teachers’ motivation to be observed in classroom observation activities.

### Perceived Value

The classroom can be understood as a shared creative community that shares certain ways of communicating while forming cognitions into a specific learning environment (Lojdová, 2019). Recent research has highlighted classroom observation activities as a way to positively change classroom practice (Ro, 2020). One study identified a gap between what teachers think they should do in the classroom and what they feel they are actually able to do (Hong et al., 2019b). To address this gap, teachers need to demonstrate their teaching to peers to get effective feedback (Hong et al., 2020). In terms of positive effects, there is a mutual learning value for the observers in classroom observation activities to evaluate and respond to teachers (Martinez et al., 2016). Similarly, in classroom observation activities, the value perception of the one who is observed and the observer changes as knowledge is constructed (Bruns et al., 2018). Teachers being mutually engaged in interaction in the classroom allows for understanding differences between being observed and observing in the classroom, which is vital to co-constructing knowledge and perspectives of teaching practice (Martinez et al., 2016). However, it is unclear whether holding constructivist teaching beliefs affects teachers’ teaching practices during classroom observations. Very little research has been conducted to examine whether being a teaching model can produce meaningful learning for participants in classroom observations.

### Psychosocial Stress

Stress is a psychosocial phenomenon (Blascovich et al., 2001) which includes more implicit and psychosocial components as well as cognitive and motivational aspects (e.g., Mendes et al., 2008). Teachers face a variety of job demands in which psychosocial aspects are fundamental because of the nature of the teaching environment (Wischlitzki et al., 2020). In classroom observation activities, stress is often heightened when it comes to meeting the demands of peers’ requests for improvement or standardized assessment items (Mourshed et al., 2010). By investigating classroom stressors and how they are related to psychosocial stress during actual teaching, this research deepens our understanding of teacher stress.

Psychosocial stress can lead teachers into a depressive mood where obvious symptoms will be exhibited such as loss of interest in the teaching activity (Mendoza-Castejon et al., 2020). It is possible that psychosocial risk factors, including self-reported depression and stress, can hinder teachers’ ability to support their students’ development in terms of both academic and socio-emotional growth (Sandilos et al., 2015; Roberts et al., 2016). High levels of depression and stress among teachers have been found to have a detrimental impact on the quality of their classroom instruction (Buettner et al., 2016; McLean et al., 2018; Bardack and Obradović, 2019). On the contrary, a previous study found that stress was associated with lower levels of demand to be observed (Bottiani et al., 2019). However, teaching effectiveness is not usually achieved under classroom observation as it puts more pressure on teachers (Taylor and Tyler, 2012). This study therefore investigated the role of psychosocial stress in teachers’ perceptions of being observed during classroom observation activities.

### Continuous Intention

If classroom observation activities are to be used to improve the quality of teaching and learning or to improve teaching skills, each observed lesson should be conducted multiple times in different classrooms (Ho and Kane, 2013). Therefore, teachers need to be willing to continuously engage in classroom observations in order to improve their teaching skills. For example, McGannon and Spence (2010) proposed that suggestions for improvement can be a psychological issue for some individuals to turn others’ comments into a process or mechanism for internal self-conceptualization. However, suggestions for improvement can lead to resistance and can affect their intention to continue taking part in observations. On the other hand, in line with the third-person effect, the feedback given to the observer through the observations of peer team members provides the observer with opportunities for continuous improvement (Conson et al., 2017). In the case of classroom observation activities, no previous research has specifically examined how teachers are influenced by the educational culture of their schools while maintaining their willingness to be observed in the context of Taiwanese educational culture. Thus, the present study aimed to understand teachers’ continuous intention to be a teaching model in classroom observations.

## Materials and Methods

The study is a cross-sectional quantitative study in which theoretical and literature-based scales were selected and a research model was constructed. A questionnaire survey was then used to collect data and conduct statistical analysis on teachers’ data to identify the relationship between cognitive and intentional factors in classroom observations.

### Research Model

**FIGURE 1:**
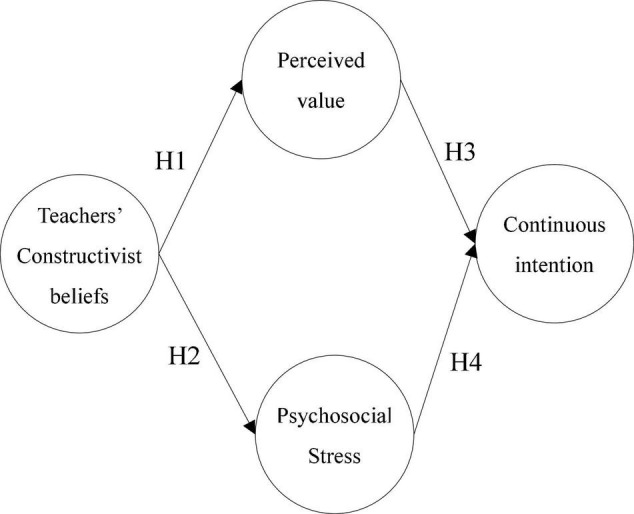
Research model.

In social cognitive theory, Bandura (1986) proposed a triadic interaction theory of causality in which behavior, personal factors (including beliefs), and environmental factors influence each other. Based on social cognitive theory of pattern behaviors, people are often motivated to be more courageous in trying to learn what they think will lead to desired outcomes based on observations of exemplars and other experiences in order to obtain outcome expectations-behavior for different learning actions and to consistently achieve their learning goals (Schunk and DiBenedetto, 2020). Accordingly, this study constructed a research model and proposed four research hypotheses to explore the relationship between teachers’ constructive beliefs, values and stressors, and their continuous intention to take the role of being observed in classroom observations, as shown in Figure 1.

### Hypotheses

#### Teachers’ Constructivist Beliefs and Perceived Values

In educational settings, social culture influences teachers’ positive or negative beliefs about teaching and learning, and these teaching beliefs then influence their teaching efforts (Sato and Oyanedel, 2019). As conducting classroom observation activities in different classrooms represents a complex and often challenging environment for teachers, teachers’ beliefs need to be valued. Each teacher’s participation in classroom observations may have different value in different teaching systems and settings (Du Plessis, 2019). For example, Martinez et al. (2016) proposed a framework for analyzing the value of classroom observations which outlines the conceptual learning of teaching practices as teachers’ conceptual value, and gathering information used to critique teachers’ performance as methodological value. That is, the feedback comments provided by the observers to the observed teachers can also result in teachers with different teaching beliefs having different value perceptions of the observed teachers (Haiyan et al., 2017). However, few studies have aimed to understand how teachers’ constructivist beliefs can affect their value perceptions of being observed in classroom observations; thus, to explore the correlation, a hypothesis was proposed as follows:

H1: Constructive beliefs of teachers are positively related to perceived value of being observed.

#### Teachers’ Constructivist Beliefs and Psychosocial Stress

It is important to note that teachers’ beliefs may have different expectations regarding the professional concepts of teaching (e.g., strategies and techniques for teaching materials) learned through classroom observation activities (Taylor and Tyler, 2012; Taut and Sun, 2014). If teachers’ beliefs about constructive oriented teaching are strong, they may actively seek professional feedback; conversely, if their beliefs about didactic-oriented teaching are strong, they may experience adverse reactions such as anxiety or stress in the assessment mechanism of classroom observations (Perera and John, in press). To date, few studies have examined the psychosocial pressure of classroom observation from the perspective of the teacher being observed, as reflected in the social cognitive learning perspective (Ro, 2020). Therefore, this study examined teachers’ constructivist beliefs to understand their perceived value or stress when being observed in classroom observation activities; the following hypothesis was therefore proposed:

H2: Teachers’ constructivist beliefs are negatively related to psychosocial stress.

#### Perceived Value and Continuous Intention

In collectivistic cultures, perceived values have a strong influence because in such societies social factors tend to serve as pertinent guides for members to engage in activities (Huang et al., 2022). In classroom observations, values are weighted differently according to the feedback received from classroom observation activities, and this weighting is judged by teachers in terms of instructional practice and effectiveness (Haiyan et al., 2017). Moreover, teachers who have stronger constructivist teaching beliefs have been shown to actually implement constructivist teaching practices which can help students to develop knowledge. The teachers are then likely to continue to engage in those behaviors (Hong et al., 2019a). However, teaching practice in classroom observation needs to be updated on the basis of the constructivist approach according to peers’ feedback to improve teachers’ motivation to participate in the classroom observation (Tas et al., 2018). Therefore, to understand the relationship between perceived value of the observed teachers and the teachers’ willingness to play the teaching models in classroom observation, the following hypothesis was proposed:

H3: Perceived value is positively related to continuous intention.

#### Psychosocial Stress and Continuous Intention

There are internal and external stressors in psychosocial pressure and adaptation (Zacharias et al., 2022). Due to insufficient knowledge and skills as an internal stressor in classroom teaching, teachers may experience incongruent job-related confidence that leads to stress or anxiety about teaching (Anderson et al., 2022). On the other hand, past research has suggested that people may have external stressors when they sense a social-evaluative threat (SET) (Woody et al., 2018). The reason for this is that teachers have to face the teaching scene alone and open up the classroom to the public; they may therefore be under the threat of social commentary and so feel anxious (Liu, 2017). These types of psychosocial stressors from the event will have a negative influence on people’s intention to continue with the activity (Gupta and Arora, 2017). Therefore, in order to understand the relationship between perceived value and psychosocial stress of the observed teachers and their willingness to assume the teaching model role in classroom observation activities, the following hypothesis was proposed:

H4: Psychosocial stress is negatively related to continuous intention.

#### Teachers’ Constructivist Beliefs and Continuous Intention

From a social self-identification perspective, in public classroom observation activities, observed teachers adjust their teaching style according to the socio-cultural context. In current educational contexts, the teaching model consisting of a didactic cycle is becoming less frequently used and is being replaced by the inquiry-oriented approach; however, a previous study suggested that teachers with more constructivist teaching beliefs are more likely to continue to engage in active and interactive teaching activities than those with didactic approaches (Tas et al., 2018). In classroom observation activities, reciprocal interaction helps participants to evaluate and give feedback on teaching problems. Teaching beliefs have both positive and negative effects on teachers’ willingness to be a teaching model in classroom observation activities (Schunk and DiBenedetto, 2020). To understand how teachers’ constructivist beliefs relate to their continuous intention to be a teaching model in classroom observations is hypothesized as follows:

H5: Teachers’ constructivist belief is positively related to continuous intention.

### Procedure

The present study adapted snowballing sampling to collect data. First, we used Google Forms to distribute online questionnaires; second, we posted the link on an Instant Message platform, Line, which is the most popular platform in Taiwan, particularly in teachers’ groups; third, information was attached requesting respondents to share the link with other Line groups. The questionnaires were collected from May 17 to June 25, 2021, with 426 questionnaires collected from full-time K-12 teachers in Taiwan.

Considering ethical issues, the present study required participants to be given information about what they were being asked to do. At the beginning of the questionnaire, they were asked for their consent, and were given the option to withdraw from the study if they so wished. They knew they were participating in an evaluation study, that their data would be treated anonymously, and that the study results could be published.

### Participants

The total number of participants in this study (number of returned questionnaires) was 426, and 77 invalid data were removed, resulting in 349 valid study participants, with a valid return rate of 81.9%, including 127 male participants and 222 female participants; 25 participants (7.1%) were 30 years of age or below, 101 (28.9%) were between 31 and 40 years of age, 148 (42.4%) were between 41 and 50 years of age, and 75 (21.5%) were over 50 years of age. The number of participants with university degrees was 87 (24.9%), 244 (69.9%) had master’s degrees, and 18 (5.2%) had doctoral degrees; 144 (41.3%) were teaching in elementary schools, 126 (36.1%) in junior high schools, and 79 (22.6%) in high schools.

### Instruments

This was a quantitative validation study, and data were collected through a questionnaire that was developed with reference to past research and related theories, and was then reviewed by experts to ensure its content validity. The revised questionnaire was given to 10 teachers who had experience of being a teaching model; they pointed out any unreadable items that needed revising to ensure face validity.

#### Constructivist Beliefs of Teachers

According to constructivist beliefs, students have control over and regulate their own learning processes. It is expected that teachers will help students build their own personal understanding and knowledge, and that they will empower their students to grow (Belo et al., 2014). Based on these concepts, we developed 10 items to measure participant teachers’ perceptions of constructivist beliefs. Examples of items include: “I think teachers should always encourage students to come up with their own answers to questions,” “I think the teaching process needs to stimulate an atmosphere of independent inquiry and interaction,” and “I believe that the teaching process needs to encourage students to acquire, interpret and organize their own knowledge obtained from experiences through active classroom participation.”

#### Perceived Value

Perceived values are among the main drivers of performing numerous value-congruent behaviors (Bouman et al., 2021). The value an individual places on something comes from the accumulation of their inner perceptual experience and affirmation from others in the group, based on their ability to creatively using their teaching skills (Cui et al., 2022). In line with this, we designed seven items to examine participants’ perceived value of being observed in classroom observations. Example items are: “I think being observed helps me to design my teaching materials more creatively,” “I think being observed helps me to design my teaching activities more logically,” and “I think that being observed helps me to practice my lesson according to different learning levels.”

#### Psychosocial Stress

The Perceived Stress Scale and the Self-Rating Depression Scale, which have been validated in Chinese to evaluate individuals’ psychosocial stress (Wang et al., 2011) were referred to in this study. Based on this concept, we designed seven items to measure participants’ perceptions of psychosocial stress during classroom observation. Example items are: “I feel pressured to be observed by other teachers” and “I am concerned that my teaching performance is not appreciated by other teachers.”

#### Continuous Intention to Be Observed

Individuals who are clearly aware of both their inner world (e.g., thoughts and emotions) and their outer world (e.g., surroundings) maintain a present-oriented consciousness, and rely on presenting at observations (e.g., Richards et al., 2018). Accordingly, we designed five items to measure participants’ continuous intention to be observed in classroom observations. Example items are: “I will actively participate if the school recommends me as a teacher to be observed in my school,” “I will actively present if there is a need for activities to be observed outside my school,” and “I will actively prepare if there is a need for a classroom observation activity.”

## Results and Discussion

### Item Analysis

In this study, first-order confirmatory factor analysis (first-order CFA) was used for item analysis. Statisticians suggest that the χ^2^/*df* value should be less than 5; therefore, the root mean square error of approximation (RMSEA) should be less than 0.1; GFI and AGFI should be higher than 0.8. The questions with a factor loading (FL) not higher than 0.5 should be deleted from the original questionnaire (Hair et al., 2010; Kenny et al., 2015). In this study, the factor loadings ranged from 0.580 to 0.839 for teachers’ constructive beliefs, 0.878–0.928 for value perception of being observed during an activity, and 0.878–0.928 for psychosocial stress of being observed during an activity. The deletion results were as follows: teachers’ constructive beliefs was reduced from 10 to 9 items; value perception of being observed during the activity, from seven to five; psychosocial stress of being observed during the activity, from seven to six; and continuous intention to be observed during the activity, from five to four, as shown in Table 1.

**TABLE 1:** First-order CFA for item analysis.

Index	Threshold value	Teachers’ constructive beliefs	Psychosocial stress	Value perception	Continuous intention
χ^2^/*df*	<5	4.17	3.80	3.32	4.20
RMSEA	<0.1	0.09	0.09	0.08	0.09
GFI	>0.8	0.93	0.96	0.98	0.98
AGFI	>0.8	0.88	0.93	0.95	0.93
FL	>0.05	0.58∼0.84	0.88∼0.93	0.68∼0.83	0.86∼0.92
*t*	>3	17.90∼23.83	27.22∼28.31	23.07∼26.96	16.69∼18.53

In this study, the external validity of the questions was used to determine the range of interpretation (Cor, 2016), and the *t*-values for the first 27% and last 27% of all respondents for each question were examined; if the *t*-value was greater than 3 (***p < 0.001), the external validity was considered significant. Table 2 shows that the *t*-values were higher than 16.687 (***p < 0.001), which indicates that all questions in this study had external validity (Green and Salkind, 2004), as shown in Table 1.

**TABLE 2:** Reliability and validity analysis.

Construct	*M*	*SD*	α	FL	CR	AVE
Teachers’ constructive beliefs	4.39	0.50	0.90	0.72	0.91	0.53
Psychosocial stress	2.58	0.89	0.96	0.91	0.96	0.82
Perceived value	3.68	0.88	0.96	0.91	0.96	0.79
Continuous intention	3.55	0.91	0.95	0.90	0.93	0.77
						

### Reliability and Validity Analysis

In this study, Cronbach’s α was used to confirm the internal consistency of the test scale, and composite reliability (CR) was used to check the reliability. Hair et al. (2010) suggested that CR values should exceed the criterion of 0.7. In this study, Cronbach’s α values ranged from 0.90 to 0.96 and CR values ranged from 0.91 to 0.96, which met the suggested criteria, as shown in Table 2.

In this study, convergent validity was determined by factor loading (FL) and average variance extracted (AVE), with Hair et al. (2010) stating that FL should be higher than 0.5 and that items below this value should be removed. The items retained in this study met the criteria suggested by scholars, with factor loadings ranging from 0.72 to 0.91 for all constructs. Hair et al. (2011) suggested that AVE values should be greater than 0.5 to represent constructs with good convergent validity. The AVE values ranged from 0.53 to 0.82, as shown in Table 2.

Awang (2015) suggested that each construct has discriminant validity if its AVE root number value is greater than the Pearson correlation coefficient value of other constructs. The results of the analysis showed that each construct in this study had discriminant validity, as shown in Table 3.

**TABLE 3:** Discriminant construct validity.

Construct	1	2	3	4
1. Teachers’ constructive beliefs	(0.73)			
2. Perceived value	0.33	(0.89)		
3. Psychosocial stress	0.29	0.38	(0.91)	
4. Continuous intention	0.34	0.33	0.55	(0.88)

*The absolute value on the diagonal is the square root of AVE, and the other values are the values of the related coefficients.*

### Model Fit Analysis

Statisticians recommend that χ^2^/*df* be less than 5 (Hair et al., 2010), RMSEA be less than 0.1, and GFI, AGFI, Normative Fit Indicator (NFI), NNFI, CFI, IFI, and RFI all be greater than 0.8 (Abedi et al., 2015). The Parsimonious Normative Fit Indicator (PNFI) and Parsimonious Goodness Fit Indicator (PGFI) should be greater than 0.5 (Hair et al., 2010). The fitted index values for this study are χ^2^ = 426.6, *df* = 248, χ^2^/*df*. = 1.72, RMSEA = 0.05, GFI = 0.91, AGFI = 0.90, NFI = 0.95, NNFI = 0.97, CFI = 0.98, IFI = 0.98, RFI = 0.94, PNFI = 0.85, and PGFI = 0.75.

### Path Analysis

**FIGURE 2:**
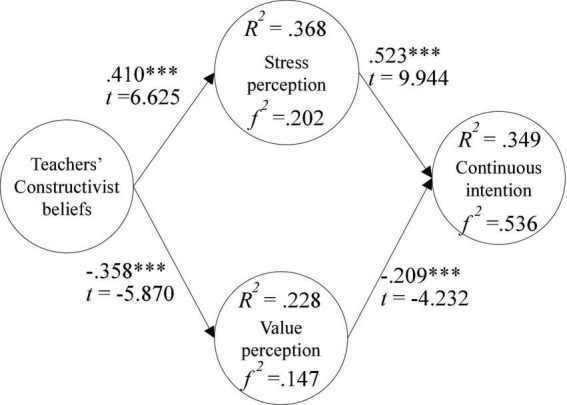
Validation of the research model. ***p < 0.001.

Model validation results showed that teachers’ constructive beliefs had a positive effect on their psychosocial stress when being a teaching model in classroom observations (β = 0.410***, *t* = 6.625), teachers’ constructive beliefs had a negative effect on value perception when being a teaching model in classroom observations (β = −0.358***, *t* = −5.870), value perception of being a teaching model in classroom observations had a positive effect on continuous intention (β = 0.523***, *t* = 9.944), and psychosocial stress from being a teaching model in classroom observations had a negative effect on continuous intention (β = −0.209***, *t* = −4.232), as shown in Figure 2.

The explanatory power of teachers’ constructive beliefs for value perception of being a teaching model in classroom observations was 36.8%, and *f*2 was 0.202. The explanatory power of constructive beliefs of teachers for psychosocial stress from being a teaching model in classroom observations was 22.8%, and *f*2 was 0.147. The explanatory power of stress and value perception of being a teaching model in classroom observations for continuous intention was 34.9%, and *f*2 was 0.536, as shown in Figure 2.

### Indirect Effect Analysis

Indirect effect analysis shows that Teachers’ Constructivist beliefs had a positive effect on their continuous intention (β = 0.289**), as shown in Table 4.

**TABLE 4:** Indirect effect analysis.

	Teachers’ constructivist beliefs	
	
	β	95% CI
Continuous intention	0.289**	[0.212, 0.359]

**p < 0.01.

### Discussion

Bandura’s SCT is based on the premise that through observation, people can learn from the events or tasks they engage in Schunk and DiBenedetto (2020). Teacher beliefs are teachers’ personal beliefs about their own learning, and having positive teaching beliefs is associated with positive beliefs about student learning (Meirink et al., 2009). From the findings of this study, participants had strong constructivist beliefs (*M* = 4.39, *SD* = 0.50). The results of the study showed that participants had positive perceived value of being observed (*M* = 3.68, *SD* = 0.88); contrary, they had low levels of psychosocial stress (*M* = 2.57, *SD* = 0.89). Continuous intention was defined as the willingness to cooperate with the implementation of educational policies (Hong et al., 2019b), and the study found that participants in this study had positive continuous intention to participate in classroom observations (*M* = 3.55, *SD* = 0.91).

#### Constructive Beliefs of Teachers Are Positively Related to Perceived Value of Being Observed

Considering the Confucian culture, conformity is a way of interpreting knowledge in educational settings; ideas of individuals are often subordinated to the ideas of the group, and members of the group usually feel more comfortable when they conform to the group ideas (Sum and Kwon, 2020). How teachers’ beliefs in being a teaching model and the effect on perceived value and psychosocial stress in relation to their continuous intention to be observed in classroom observations in a Confucian culture context was hypothesized and verified as follows.

Constructivist teaching involves encouraging students to use their imaginations, and fostering creative thinking through ingenious methods of transmitting the learning content (Sum and Kwon, 2020). However, social culture influences teachers’ positive or negative beliefs about teaching and learning, and teaching beliefs influence their teaching efforts (Sato and Oyanedel, 2019). Each teacher’s participation in classroom observation may have different values in different teaching systems and settings (Du Plessis, 2019). For example, Martinez et al. (2016) proposed a framework for analyzing the value of classroom observation which outlined the conceptual learning of teaching practices for teachers’ conceptual value, and gathering information used to critique teaching practices as methodological value. Considering the value of classroom observation, Hong et al. (2019a) argued that teachers’ teaching beliefs do not necessarily perfectly align with their actual teaching practices in the classroom; however, if teachers’ beliefs about constructive approaches are strong, they may actively seek professional feedback; if their beliefs about didactic approach are strong, they may perceive the value of classroom observation as being low (Perera and John, in press). In this study, how teachers’ constructivist beliefs affect their value perception of being observed in classroom observations was examined, and H1 was positively verified.

#### Teachers’ Constructivist Beliefs Are Negatively Related to Psychosocial Stress

Sato and Oyanedel (2019) suggested that in educational settings, social culture influences teachers’ positive or negative beliefs about teaching and learning, and that teaching beliefs also influence teachers’ stress when teaching. Drawing on the social cognitive learning perspective, Ro (2020) suggested that the psychosocial pressure of classroom observation occurred from the perspective of the teacher being observed. If teachers’ constructive beliefs are weak, they may react negatively to the assessment mechanisms in classroom observation, such as by experiencing stress (Perera and John, in press). Considering that Taiwan’s culture is grounded in Confucianism, in pursuit of a harmonious relationship, Chinese teachers tend to conform to collective norms, which can avoid conflict in learning activities (Misco and Tseng, 2018; Huang et al., 2020) and reduce their psychosocial stress (So and Hu, 2019). However, the results of this study showed that the higher level of constructive beliefs participants had, the lower level of psychosocial stress from being observed they would have in classroom observations, indicating that H2 was negatively supported.

#### Perceived Value Is Positively Related to Continuous Intention

The influence of perceived values is likely to be stronger in collectivistic cultures due to the fact that social factors tend to serve as pertinent guides for members to engage in activities in such cultures (Huang et al., 2022). In classroom observations, values are weighted differently according to the feedback received from classroom observation activities, and this weighting is judged by teachers in terms of instructional practice and effectiveness (Haiyan et al., 2017). Moreover, teachers with stronger constructivist teaching beliefs have been found to actually adopt constructivist teaching practices which can help students develop their knowledge; the teachers are then likely to continue to adopt those behaviors (Hong et al., 2019a). Future motivation implies behaviors derived from an individual’s values; when value is high, there is continuing attendance and performance (Pownall, 2012). Therefore, to understand the relationship between the perceived value of the teacher who is observed and the teacher’s willingness to be the teaching model in classroom observation, H3 was positively supported.

#### Psychosocial Stress Is Negatively Related to Continuous Intention

Psychosocial adaptation is a continuous process involving internal and external interactions while facing stress in a particular situation (Zacharias et al., 2022). The internal stressor is that when teachers have adaptive beliefs, it is possible that there is incongruence with their classroom practices because of a lack of knowledge and skills, and of job-related confidence that can lead to stress or anxiety about teaching (Anderson et al., 2022). The other external stressor is that they sense a social-evaluative threat (SET) (Woody et al., 2018). Teachers may be under the threat of social commentary and so feel anxious, which may prevent them from participating in the observation with the intent of sharing their teaching practices (Liu, 2017). Psychosocial stress from the event will negatively influence people’s intention to continue engaging in the event (Gupta and Arora, 2017). Liu et al. (2022) pointed out that teachers in Chinese culture can experience psychosocial stress due to face-saving and hiding their true opinions so as to foster a harmonious communication process. Therefore, to understand the relationship between perceived value and psychosocial stress of the observed teachers and their willingness to assume the role of observer in classroom observations, H4 was negatively verified.

#### Teachers’ Constructivist Belief Is Positively Related to Continuous Intention

From a social self-identification perspective, in public classroom observation activities, observed teachers adjust their teaching style according to the socio-cultural context. A previous study suggested that teachers with more constructivist teaching beliefs are likely to continue to engage in active and interactive teaching activities (Tas et al., 2018). Moreover, in classroom observation activities, reciprocal interaction helps participants to evaluate and give feedback on teaching problems. Teaching beliefs have both positive and negative effects on teachers’ willingness to be teaching models in classroom observation activities (Schunk and DiBenedetto, 2020). Based on the third-person effect, the feedback given to the observer through the observations of peer team members provides the teacher who is being observed with opportunities for continuous improvement (Conson et al., 2017). H5, which was proposed to understand how teachers’ constructivist beliefs related to continuous intention to be the observed teaching model in classroom observations, was positively verified.

## Conclusion and Recommendations

### Conclusion

Classroom observation activities are used as an important tool for teacher development and evaluation. However, classroom observation activities may cause stress and fear among teachers, and feedback suggestions may lead to individual resistance or further self-protective measures. Therefore, teachers’ perceptions of the value of classroom observation and psychosocial stress when participating in activities are worth exploring. Accordingly, in this study we constructed a research model based on social cognitive theory to explore teachers’ continuous intention to be observed in classroom observations. The results revealed that teachers’ constructivist beliefs can positively predict their perceived value, but negatively predict their psychosocial stress, while their continuous intention can be positively predicted by perceived value, but negatively predicted by psychosocial stress. Finally, teachers’ constructivist beliefs can positively predict their continuous intention to be observed.

### Implications

Most of the current research focuses on the benefits to teachers’ professional development and students’ learning outcomes, but little research has examined the psychological perceptions of teachers from a socio-cognitive-psychological perspective and the continuous intention to participate in classroom observations. However, these are important factors that affect the quality of classroom observation activities in Confucian culture.

Although classroom observation activities are considered to be an integral component of teacher development and evaluation, in general, teachers who are observed as a teaching model do not have a high level of continuous intention. From a policy-acceptance perspective, the implementation of classroom observations should be based on the high perceived value and low psychosocial stress of teachers for the development of classroom observation. The results of this study can provide a reference for professional development program designers to consider how to improve classroom observations.

Additionally, the willingness of participants is one of the most important and critical factors affecting policy implementation. Referring the result of this study, the policy planners and administrators should consider what affected teachers’ social cognitive learning before implementing the promotion of classroom observation. At the same time, incentive mechanisms may be implemented to encourage participants to achieve the purpose of this policy implementation.

### Future Study

This study revealed that elementary school teachers were less likely to be subjected to classroom observation than secondary school and special classroom teachers, and that teachers perceived classroom observation activities as more of an assessment than as feedback for teacher professional development than did principals (Lam, 2001). It is evident that teachers at different levels of education have different perceived values of classroom observation and psychosocial stress in their teaching model roles. However, this part of the study was not explored in the present paper, so in subsequent articles, we can analyze the differences in teachers’ perceptions at different levels of education to explore more deeply the perceived value and psychosocial stress in different types of classroom observations.

Teacher self-efficacy (teachers’ belief that they can change the way students learn) is one of the most studied aspects of classroom settings (Miller et al., 2017), given that self-efficacy has a positive impact on all aspects of teacher practice (Moradkhani et al., 2017). By exploring teachers’ self-efficacy with respect to perceived value and psychosocial stress of classroom observation, it should be possible to include the self-efficacy factors in the research model for examination in follow-up studies.

## Data Availability Statement

The raw data supporting the conclusions of this article will be made available by the authors, without undue reservation.

## Ethics Statement

Ethical review and approval was not required for the study on human participants in accordance with the local legislation and institutional requirements. Written informed consent for participation was not required for this study in accordance with the national legislation and the institutional requirements.

## Author Contributions

P-HC, J-CH, and J-HY: concept and design, drafting of the manuscript, critical revision of the manuscript, acquisition of data, and statistical analysis. P-HC, J-CH, and Y-JH: critical revision of the manuscript. All authors contributed to the article and approved the submitted version.

## Conflict of Interest

The authors declare that the research was conducted in the absence of any commercial or financial relationships that could be construed as a potential conflict of interest.

## Publisher’s Note

All claims expressed in this article are solely those of the authors and do not necessarily represent those of their affiliated organizations, or those of the publisher, the editors and the reviewers. Any product that may be evaluated in this article, or claim that may be made by its manufacturer, is not guaranteed or endorsed by the publisher.
